# Association between poor sleep quality and an increased risk of dry eye disease in patients with obstructive sleep apnea syndrome

**DOI:** 10.3389/fmed.2022.870391

**Published:** 2022-10-31

**Authors:** Qi Pu, Zhen Wu, Ao-Ling Li, Xiao-Xiao Guo, Jing-Jie Hu, Xin-Yu Li

**Affiliations:** ^1^Department of Ophthalmology, Tongji Hospital, Tongji Medical College, Huazhong University of Science and Technology, Wuhan, China; ^2^Department of Ear, Nose, and Throat, Changshu No. 2 People’s Hospital, Changshu, China

**Keywords:** obstructive sleep apnea, dry eye disease, sleep quality, a case-control study, dry eye

## Abstract

**Purpose:**

Obstructive sleep apnea (OSA) is related to an increased incidence of dry eye disease (DED). However, their exact relationship is unknown and requires further well-designed studies with advanced mechanisms detection.

**Patients and methods:**

This case–control study included 125 OSA cases and 125 age–gender-matched controls enrolled in the hospital between 1 January and 1 October 2021. OSA diagnosis and classification were performed using a polysomnography (PSG) assay. Detailed ophthalmological examinations, including the Schirmer I test, corneal staining, and ocular surface disease index (OSDI), were used to detect DED-related parameters. A comprehensive ocular surface assay was performed to measure a series of parameters, including first non-invasive first tear film break-up time (f-NIBUT), average non-invasive first tear film break-up time (av-NIBUT), tear meniscus height (TMH), and loss of meibomian gland. In addition, the Pittsburgh Sleep Quality Index (PSQI) scale was used to assess sleep quality.

**Results:**

Compared to the control, the OSA group showed an increased DED risk (*P* = 0.016) along with an increased PSQI score and a higher rate of poor quality sleep (*P* < 0.001 and *P* = 0.007, respectively). Stratification of OSA cases indicated that DED-related parameters were impaired in patients with severe OSA (*P* < 0.05). The analysis of DED-parameters-related factors showed significant correlations between OSA-related indexes and PSQI (*P* < 0.05). Moreover, the poor sleep quality group in the OSA cases showed worse DED-related parameters (*P* < 0.05), which was not observed in the control group.

**Conclusion:**

OSA, especially the severe stage OSA, was related to an increased risk of DED. Also, sleep quality was correlated with the onset of both OSA and DED, where poor sleep quality revealed a relationship between OSA and the risk of DED. Overall, our findings provided evidence for advanced management of DED and OSA in future.

## Introduction

Obstructive sleep apnea (OSA) syndrome is mainly manifested as snoring during sleep, accompanied by apnea and superficial breathing. OSA cases include the repeated occurrence of hypoxemia, hypercapnia, and sleep structure disorders at night, along with well-accepted related damages, such as cardiovascular, cerebrovascular, and metabolic dysfunctions (1). Understanding the potential pathological contributions of OSA to cancers, depression, and asthma can also update our knowledge of its biological effects (2, 3). In addition, a previous cross-sectional study involving 3,303 subjects showed that OSA was an independent risk factor for dry eye disease (DED) (4). Therefore, detecting the relationship between OSA and DED may provide information to manage both diseases.

Dry eye disease is regarded as one of the most common public health problems, with a prevalence rate of 5 to 50% (5). It leads to various symptoms, including a sensation of the presence of a foreign body, itching, and a burning sensation, and involves both physical and emotional discomforts described in ophthalmology (6). Currently, the management of DED includes eye drops targeting tear dysfunction, as well as abnormal inflammation in the ocular surface. However, the overall satisfaction degree for DED treatment is unsatisfactory (7). To provide better preventive and therapeutic strategies for DED patients, it is necessary to identify the risk factors of DED. Since OSA is a potentially harmful factor of DED, targeting OSA might provide advanced improvements in the management of DED. A study including both severe and mild/moderate OSA cases showed that OSA influenced meibomian gland alterations (8). Since meibomian gland dysfunction (MGD) is one of the structural bases of DED, OSA can be considered to harm the functioning of the meibomian gland, promoting the incidence of DED. Although OSA is relatively accepted as a potentially harmful risk factor for DED, its detailed pathological process remains unclear.

Mounting evidence demonstrates that sleep disorders are becoming prevalent health issues worldwide, with an estimate of 150 million people suffering from them (9). OSA is one of the most detected sleep disorders causing excessive daytime sleepiness along with other potential sleep disorders (10). Yet, about 80% of moderate and severe OSA cases remain undiagnosed (11). Hence, the potential influences of poor sleep quality caused by OSA should be investigated by further advanced wide-range studies. Although sleep disorders are prominent, they are overlooked as serious comorbidities of DED, and there is also a lack of information on their detailed pathological connection (12). As poor sleep quality is related to both OSA and DED, we conjecture that poor quality of sleep might contribute to DED in patients with OSA. To verify this hypothesis, we conducted a case–control study with the following purposes: (1) recording and analyzing the detailed DED-related parameters and sleep quality among the OSA cases and age–gender-matched controls, and (2) detecting the association between poor sleep quality and DED incidence in OSA cases.

## Materials and methods

### Study design and participants

**FIGURE 1:**
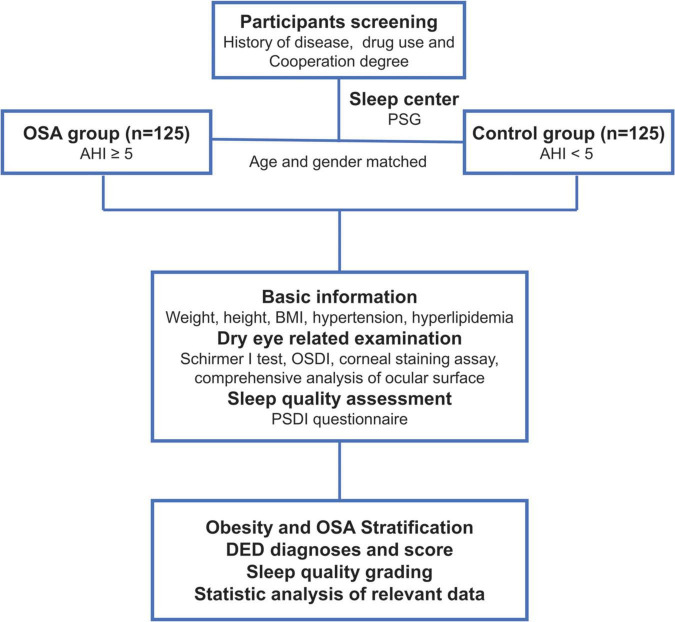
Detailed flow diagram of study design and participants screening.

We conducted a hospital-based, case–control study involving OSA cases and healthy controls from Changshu Hospital between 1 January and 1 October 2021. The included participants were screened consecutively based on their history of the disease, drug use, and cooperation during the study period. After performing the polysomnography (PSG) examination in the Sleep Center, a total of 125 OSA cases and 125 age–gender-matched controls were included in the advanced experiments. The general information, DED-related examination records, and sleep quality assessments of all participants were collected, and after a series of processing, including OSA stratifications, DED scores, and sleep quality classifications, the data were used for statistical analyses. The detailed flow diagram of the study design and screening of participants is presented in Figure 1.

This case–control study was carried out as per the Declaration of Helsinki and was approved by the Institutional Review Board and Ethics Committee of the Changshu No. 2 People’s Hospital. Furthermore, written informed consent was obtained from all included participants.

The inclusion criteria included OSA cases confirmed by PSG, while the control group included the age–gender-matched participants without OSA. The age difference between OSA cases and matched controls was less than 2 years.

The exclusion criteria included OSA cases and relevant controls based on our team’s related publications with slight modifications (13, 14). Participants were excluded from the OSA group if they met any of the following criteria: (1) patient with heart diseases, cerebrovascular diseases, autoimmune disorders, metabolic syndromes, malignant tumors, and psychological disorders; (2) patient with lacrimal diseases, systemic Sjogren’s syndrome, or chemical injury in the cornea and conjunctiva; (3) history of the previous ocular surface, vitreoretinal, lacrimal canal, and tear gland surgeries in the recent 6 months; (4) patients with allergies or other conditions not able to finish the examination or treatments; (5) recent eye drops usage within 1 month; (6) history of sinusitis-nasal septum deviation and other diseases affecting sleep; (7) recently rotated night shift work for at least past 1 week; and (8) unwillingness to participate in the research.

### Polysomnography examination

After performing a PSG examination using the Respironics Alice 5 system at the Sleep Center of the Department of Ear-Nose-Throat in Changshu No. 2 Hospital, all participants were divided into OSA and control groups. All detection procedures followed our previous research (13). The operation protocol included monitoring of at least 7 h of sleep time according to the patient’s sleep habits, accompanied by the usage of PSG to monitor standard 3-lead electrocardiogram (EEG), electromyography (EMG) signal acquisition of heartbeat, eye movement, jaw movement, and leg movement. While the respiratory movement was automatically identified using the chest and abdomen respiratory induction plethysmography (RIP) belt, the respiratory airflow was intelligently identified by measuring the vitality and duration of the breath airflow in the nose and mouth. Subsequently, the following indicators were recorded: oral and nasal airflow; pulse saturation O_2_ (SpO_2_); sleep stages and the percentage of time in each period; the number of awakenings and other indicators; apnea-hypopnea index (AHI); obstructive apnea index (OAI); minimum blood oxygen saturation (lowest oxygen saturation, LSpO_2_); oxygen desaturation index (ODI); mean oxygen saturation (MSpO2) and percentage of time with SpO_2_ < 90% (CT90%). On the day of the PSG examination, participants were not allowed to consume coffee/strong tea, alcohol, sedative drug, or sleep during the daytime. The cutoff value of OSA diagnosis was set at AHI = 5. A value of AHI 5–15, 15–30, and ≥30 events/hour indicated mild, moderate, and severe OSA cases, respectively (15).

### Dry eye disease-related examinations

The basal DED detections were combined with a comprehensive inspection of the ocular surface to carry out DED-related examinations. The detailed process was modified based on the previous workflow of our recent publication (14), while DED was diagnosed based on non-invasive first tear film break-up time. Simultaneously, both f-NIBUT and av-NIBUT were measured as well. In addition, TMH and meibomian gland loss were detected using an Ocular Surface Comprehensive Analyzer (Sirius; CSO, Florence, Italy). The patient was instructed to place the mandible on the chin support while the forehead was maintained close to the forehead support, keeping their eyes open until the next blink. The instrument automatically captured the tear film image and displayed the measured values. The values from two repeats were used to obtain the average value. To a certain extent, TMH reflected the amount of tear secretion (Supplementary Figure 1). The Ocular Surface Comprehensive Analyzer was used to obtain a non-invasive TMH value by manually measuring the length between the corresponding points in the automatically presented tear film image. Sirius Anterior Segment Analysis System was used to obtain the imaging of upper and lower meibomian glands using the meibomian photography mode (Supplementary Figure 2). After capturing at least five images from the conjunctival surface of the upper and lower tarsal plates, the operator chose the clearest meibomian gland structure image and marked its boundary with equipment software. The device calculated the loss of the meibomian gland, and the results were classified according to the rate of loss. The mean values of two repeats were used for the final analyses.

Next, DED diagnosis and severity classification were performed using DED-related examinations, including the Schirmer I test, corneal fluorescein staining, and ocular surface disease index (OSDI) scales. The detailed operation process was achieved based on a previous publication (14). Specifically, a folded Schirmer paper strip (5 mm × 35 mm) was placed at the outer third lower eyelid margin for 5 min while minutes and the wetting length of Schirmer paper were recorded as the Schirmer scores. The Oxford corneal staining score was used in the corneal fluorescein scale, and the ocular surface was divided into three parts, namely nasal conjunctiva, temporal conjunctiva, and cornea. Each part was scored from light to heavy, with points ranging between 0 and 5. The total score ranging between 0 and 15 points was used for the final analyses. Finally, DED was diagnosed based on the Expert Consensus on Clinical Management of Dry Eye (2020) (16).

### Sleep quality assessment

The sleep quality of all participants was assessed using the Pittsburgh Sleep Quality Index (PSQI) scale (17). The scale was compiled by Buysse of the University of Pittsburgh Medical Center in 1989, which included 19 self-evaluations and five other evaluation items. The items included seven components, namely subjective sleep quality, sleep duration, sleep latency, sleep efficacy, sleep disturbance, sleep aid medication, and sleep-related daytime problems. Each dimension was scored by a 4-level scoring method, that is, between 0 and 3 points, while the total score was calculated between 0 and 21 points. Based on a previous publication by Magno et al. (18), a PSQI score of <6 points indicated good sleep quality, while a PSQI score of ≥6 points indicated sleep disorder. The higher the PSQI score, the worse the sleep quality.

### Statistical analysis

The statistical analysis was conducted using GraphPad Prism 8 software (GraphPad Software Inc., San Diego, CA, USA). The categorical variables were presented using frequency, while the continuous variables were expressed as mean ± standard deviation (SD). The Kolmogorov–Smirnov test was used to verify the normality of values, while the chi-square test was used to compare the differences in categorical variables among different groups. The *t*-test was used to compare the differences between two groups, while the analysis of variance (ANOVA) was used to compare the differences among multiple groups. For the *post-hoc* analyses of ANOVA, the Student–Newman–Keuls (SNK) method was used. The correlation analyses between OSA-related parameters, PSQI scores, and DED-related parameters were calculated using Pearson’s correlation coefficient. The difference was considered statistically significant if the *p*-value was less than 0.05.

## Results

### Baseline characteristics

Table 1 presents the baseline information, including demographics, OSA-related parameters, clinic characteristics, and sleep quality, of the participants in both OSA and control groups. Since it was an age–gender-matched case–control study, there was consistency in the status of age and indiscriminate gender distribution between OSA and relevant control groups (*P* = 0.809 and *P* = 0.552, respectively). To obtain OSA-related parameters, all participants were divided into two groups, namely OSA and control groups. Compared to the control groups, the OSA group showed significantly increased values of OSA-related parameters, including AHI, OAI, ODI, MSpO_2_, LSpO_2_, and CT90. Advanced analyses of the clinical characteristics showed higher incidences of hypertension and hyperlipidemia in the OSA cases (*P* < 0.05) along with an increased BMI value (*P* < 0.001). However, no significant difference was observed in the incidence of diabetes (*P* = 0.105). The OSA group showed a significantly higher incidence rate of DED (*P* = 0.016) than the control. Since sleep quality was reported to be related to OSA status, we examined the sleep quality in both OSA and relevant control groups using the PSQI score, which showed an increase in the PSQI score and a higher incidence rate of poor sleep quality in the OSA group (*P* < 0.05). Moreover, the detailed descriptions of PSQI contents in the OSA group showed higher scores in subjective sleep quality, sleep duration, sleep disturbance, sleep aid medication, and sleep-related daytime problems (*P* = 0.004, 0.010, 0.076, 0.002, 0.022, and 0.014, respectively).

**TABLE 1:** Demographics, clinical characteristics, OSA-related parameters, DED incidence, and sleep quality in OSA cases and relevant controls.

Variables	Control (*n* = 125)	OSA (*n* = 125)	*P*
**Demographics**			
Age (year)	53.72 ± 13.03	54.12 ± 12.99	0.809
Gender (no. of female, %)	48 (38.4)	48 (38.4)	0.552
**OSA related parameters**			
AHI (events/h)	1.58 ± 1.15	26.79 ± 17.07	**<0.001**
OAI (events/h)	0.40 ± 0.14	8.95 ± 8.55	**<0.001**
ODI (events/h)	3.30 ± 1.11	22.73 ± 15.05	**<0.001**
MSpO2 (%)	96.38 ± 1.05	94.02 ± 1.28	**<0.001**
LSpO2 (%)	90.17 ± 1.89	80.84 ± 4.21	**<0.001**
CT90 (%)	0.11 ± 0.05	4.91 ± 5.68	**<0.001**
**Clinical characteristics**			
Hypertension (n, %)	22 (17.6)	54 (43.2)	**<0.001**
Diabetes (n, %)	35 (28.0)	47 (27.6)	0.105
Hyperlipidemia (n, %)	26 (20.8)	44 (35.2)	**0.016**
BMI (kg/m^2^)	26.58 ± 4.85	30.36 ± 5.46	**<0.001**
DED (n, %)	71 (56.8)	53 (42.4)	**0.016**
**Sleep quality**			
PSQI score	6.36 ± 3.77	8.10 ± 4.21	**<0.001**
Poor sleep quality (PSQI > 5, n, %)	80 (64.0)	58 (46.4)	**0.007**
Components of PSQI			
Subjective sleep quality	0.88 ± 0.88	1.20 ± 0.95	**0.004**
Sleep duration	0.89 ± 0.85	1.16 ± 0.88	**0.010**
Sleep latency	0.99 ± 1.00	1.08 ± 0.91	0.432
Sleep efficacy	0.86 ± 0.83	1.06 ± 0.98	0.076
Sleep disturbance	0.82 ± 0.81	1.15 ± 0.92	**0.002**
Sleep aid medication	0.84 ± 0.84	1.09 ± 0.86	**0.022**
sleep-related daytime problems	1.08 ± 0.91	1.36 ± 0.95	**0.014**

Comparison among groups was calculated using unpaired *t*-test and chi-square exact test. The comparisons with statistical differences were marked in bold.

### Dry eye disease incidence and dry eye disease-related parameters in the obstructive sleep apnea and relevant control groups

Since our results suggested a higher DED incidence rate in the OSA group, the stratification of OSA groups provided a further detailed description of DED-related parameters. According to the AHI value of 5, 15, and 30 events/h, OSA cases were divided into mild, moderate, and severe groups, respectively. Generally, most DED-related parameters were significantly different in mild, moderate, and severe group between control and OSA cases; however, no significant difference was detected in their TMH values. The classic DED-related indexes, including Schirmer I test values, Oxford corneal staining score and OSDI, comprehensive ocular surface assay parameters, including f-NITBUT, av-NITBUT, TMH, and loss on eyelid meibography, can provide a better understanding of the relationship between OSA and DED. Generally, no significant difference was observed in any DED-related parameters between the mild/moderate OSA and control groups. Interestingly, most DED-related parameters were impaired in severe OSA cases, with no significant difference in Oxford corneal staining score and TMH between the control and severe OSA cases (*P* = 0.0780 and 0.064, respectively). The detailed data are presented in Table 2.

**TABLE 2:** Incidence of DED, DED-related parameters, and comprehensive analysis of ocular surface among OSA cases in different stages and the relevant controls.

Variables	Control	OSA cases			*P*
		Mild OSA	Moderate OSA	Severe OSA	
DED incidence	53 (42.4)	13 (40.6)	30 (55.6)	28 (71.8)	**0.007**
Schirmer I test (mm/5 min)	15.61 ± 6.69	15.66 ± 6.68	15.17 ± 6.99	10.00 ± 6.56	**<0.001**
Oxford corneal staining score	0.70 ± 1.01	0.25 ± 0.43	0.39 ± 1.06	1.13 ± 1.45	**0.002**
OSDI	15.92 ± 10.09	15.41 ± 11.89	18.83 ± 13.94	33.10 ± 19.58	**<0.001**
f-NITBUT (s)	7.31 ± 2.39	7.25 ± 1.60	6.79 ± 1.99	5.85 ± 2.09	**0.003**
av-NITBUT (s)	10.55 ± 3.06	10.41 ± 1.89	10.08 ± 2.25	8.90 ± 2.50	**0.011**
TMH (mm)	0.38 ± 0.07	0.38 ± 0.07	0.38 ± 0.08	0.35 ± 0.08	0.588
Loss on upper eyelid meibography (%)	17.82 ± 8.44	17.49 ± 6.10	21.12 ± 10.15	26.20 ± 11.72	**<0.001**
Loss on lower eyelid meibography (%)	19.50 ± 8.52	18.69 ± 5.76	22.02 ± 9.36	27.87 ± 12.45	**<0.001**

Comparison among groups was calculated using unpaired *t*-test and chi-square exact test. The comparisons with statistical differences were marked in bold.

### The correlations between general information, obstructive sleep apnea-related parameters, sleep quality, and dry eye disease-related parameters

Dry eye disease incidence and its related parameters were found to be significantly related to the risk and severity of OSA. In this case–control study, age and BMI were significantly associated with loss of lower eyelid meibography and Schirmer I test values, respectively (*P* < 0.05). The relationship analyses between DED-related parameters and OSA-related indexes, including AHI, OAI, ODI, MSpO_2_, LSpO_2_, and CT90, revealed a comprehensive correlation between them. Finally, the potential correlations between sleep quality and DED-related parameters were also analyzed, where sleep quality was assessed using the total PSQI score. The correlation analyses performed using the PSQI score showed negative linear correlations among Schirmer I test, f-NITBUT, av-NITBUT, and TMH values. However, positive correlations were detected among Oxford corneal staining score, OSDI score, loss on upper eyelid meibography, and lower eyelid meibography. The detailed correlation data are shown in Table 3.

**TABLE 3:** Correlation analyses of DED-related ophthalmologic parameters and relevant factors, including general information, OSA parameters, and sleep quality among all the participants.

	Schirmer I test (mm/5 min)	Oxford corneal staining score	OSDI	f-NITBUT (s)	av-NITBUT (s)	TMH (mm)	Loss on upper eyelid meibography (%)	Loss on lower eyelid meibography (%)
Age	*r* = 0.04 (−0.08∼0.17) *P* = 0.505	*r* = −0.44 (−0.17∼0.08) *P* = 0.492	*r* = −0.06 (−0.19∼0.06) *P* = 0.316	*r* = −0.01 (−0.13∼0.11) *P* = 0.873	*r* = 0.04 (−0.09∼0.16) *P* = 0.562	*r* = 0.03 (−0.09∼0.16) *P* = 0.619	*r* = 0.13 (0.01∼0.25) ***P* = 0.039**	*r* = −0.04 (−0.06∼0.18) *P* = 0.327
BMI	*r* = −0.15 (−0.27∼−0.02) ***P* = 0.02**	*r* = 0.06 (−0.07∼0.18) *P* = 0.344	*r* = 0.09 (−0.04∼0.21) *P* = 0.172	*r* = −0.07 (−0.19∼0.06) *P* = 0.302	*r* = −0.11 (−0.23∼0.02) *P* = 0.099	*r* = −0.04 (−0.17∼0.08) *P* = 0.502	*r* = 0.10 (−0.02∼0.22) *P* = 0.135	*r* = 0.09 (0.01∼0.26) ***P* = 0.031**
AHI	*r* = −0.28 (−0.39∼−0.17) ***P* < 0.001**	*r* = 0.15 (0.03∼0.27) ***P* = 0.016**	*r* = 0.44 (0.34∼0.54) ***P* < 0.001**	*r* = −0.27 (−0.38∼−0.15) ***P* < 0.001**	*r* = −0.24 (−0.35∼−0.12) ***P* < 0.001**	*r* = −0.15 (−0.27∼−0.02) ***P* = 0.019**	*r* = 0.33 (0.21∼0.44) ***P* < 0.001**	*r* = 0.42 (0.23∼0.45) ***P* < 0.001**
OAI	*r* = −0.29 (−0.40∼−0.17) ***P* < 0.001**	*r* = 0.21 (0.09∼0.32) ***P* = 0.001**	*r* = 0.47 (0.37∼0.56) ***P* < 0.001**	*r* = −0.29 (−0.40∼−0.18) ***P* < 0.001**	*r* = −0.27 (−0.38∼−0.15) ***P* < 0.001**	*r* = −0.18 (−0.29∼−0.05) ***P* = 0.005**	*r* = 0.36 (0.24∼0.46) ***P* < 0.001**	*r* = 0.45 (0.26∼0.47) ***P* < 0.001**
ODI	*r* = −0.29 (−0.40∼−0.17) ***P* < 0.001**	*r* = 0.18 (0.06∼0.30) ***P* = 0.004**	*r* = 0.42 (0.32∼0.52) ***P* < 0.001**	*r* = −0.27 (−0.38∼−0.15) ***P* < 0.001**	*r* = −0.24 (−0.35∼−0.12) ***P* < 0.001**	*r* = −0.16 (−0.28∼−0.03) ***P* = 0.013**	*r* = 0.32 (0.20∼0.42) ***P* < 0.001**	*r* = 0.42 (0.19∼0.42) ***P* < 0.001**
MSpO_2_	*r* = 0.23 (0.11∼ 0.34) ***P* < 0.001**	*r* = −0.08 (−0.21∼0.04) *P* = 0.19	*r* = −0.33 (−0.44∼0.22) ***P* < 0.001**	*r* = 0.17 (0.04∼0.28) ***P* = 0.009**	*r* = 0.14 (0.01∼0.26) ***P* = 0.03**	*r* = 0.16 (0.03∼0.28) ***P* = 0.012**	*r* = −0.27 (−0.38∼−0.15) ***P* < 0.001**	*r* = 0.29 (−0.40∼−0.17) ***P* < 0.001**
LSpO2	*r* = 0.22 (0.10∼ 0.34) ***P* < 0.001**	*r* = −0.09 (−0.21∼ 0.04) *P* = 0.165	*r* = −0.33 (−0.43∼−0.21) ***P* < 0.001**	*r* = 0.19 (0.07∼0.31) ***P* = 0.003**	*r* = 0.16 (0.04∼0.28) ***P* = 0.011**	*r* = 0.11 (−0.02∼0.23) *P* = 0.086	*r* = 0.27 (−0.38∼−0.15) ***P* < 0.001**	*r* = −0.41 (−0.39∼−0.16) ***P* < 0.001**
CT90	*r* = −0.29 (−0.40∼−0.17) ***P* < 0.001**	*r* = 0.24 (0.12∼0.36) ***P* < 0.001**	*r* = 0.43 (0.33∼0.53) ***P* < 0.001**	*r* = −0.26 (−0.37∼−0.14) ***P* < 0.001**	*r* = −0.23 (−0.34∼−0.11) ***P* < 0.001**	*r* = −0.13 (−0.25∼−0.01) ***P* = 0.035**	*r* = 0.27 (0.15∼0.38) ***P* < 0.001**	*r* = 0.38 (0.17∼0.40) ***P* < 0.001**
Total PSQI Score	*r* = 0.31 (−0.42∼−0.19) ***P* < 0.001**	*r* = 0.25 (0.13∼0.36) ***P* < 0.001**	*r* = 0.47 (0.36∼0.56) ***P* < 0.001**	*r* = −0.31 (−0.42∼−0.19) ***P* < 0.001**	*r* = −0.26 (−0.38∼−0.15) ***P* < 0.001**	*r* = −0.26 (−0.38∼−0.14) ***P* < 0.001**	*r* = 0.37 (0.26∼0.47) ***P* < 0.001**	*r* = 0.04 (0.27∼0.48) ***P* < 0.001**

The linear correlations with a significant difference were marked in bold. OSA, obstructive sleep apnea; AHI, apnea-hypopnea index; OAI, obstructive apnea index; MSpO2, mean nocturnal oxygen saturation; ODI, oxygen desaturation index; LSpO2, lowest oxygen saturation; TS90%, time spent below oxygen saturation of 90%.

### Dry eye disease-related parameters of good and poor sleep groups belonging to obstructive sleep apnea and relevant control cases

As sleep quality was related to both OSA status and DED incidence, advanced analyses were used to detect the potential contributors of sleep quality in OSA-related DED incidence. Based on the PSQI score, the participants were further divided into good sleep quality and poor sleep quality groups. Interestingly, no significance was observed in any DED-related parameters between good sleep and poor sleep groups within the control participants (*P* > 0.05). However, in the poor sleep group in the OSA cases, a significantly increased incidence of DED was observed (*P* = *0.002*). Advanced analyses included detailed classic DED-related parameters, including the Schirmer I test, corneal staining assay, and OSDI scores, which showed that OSA patients with poorer sleep tended to demonstrate less tear production and severe ocular surface damage with ocular discomfort (*P* < 0.05). Loss on both upper and lower eyelid meibography represented the meibomian gland structures and their potential biological function. Compared to the OSA cases with good sleep, the poor sleep ones demonstrated an increased rate of loss in eyelid meibography (*P* < 0.001). Table 4 presents DED-related parameters of good and poor sleep groups belonging to the OSA cases and relevant controls.

**TABLE 4:** Dry eye disease (DED)-related parameters in good and poor sleep groups of OSA cases and relevant controls.

	Control			OSA		
	Good sleep (*n* = 67)	Poor sleep (*n* = 58)	*P*	Good sleep (*n* = 45)	Poor sleep (*n* = 80)	*P*
DED (n, %)	31 (46.3)	22 (37.9)	0.370	17 (37.8)	54 (67.5)	**0.002**
Schirmer I test (mm/5 min)	15.69 ± 6.97	15.52 ± 6.36	0.889	15.81 ± 5.34	15.72 ± 7.52	**0.001**
Oxford corneal staining score	0.76 ± 0.95	0.62 ± 1.08	0.444	0.09 ± 0.29	0.43 ± 0.85	**<0.001**
OSDI	15.71 ± 9.16	16.16 ± 11.05	0.806	12.31 ± 6.41	19.13 ± 14.36	**<0.001**
f-NITBUT (s)	7.29 ± 2.44	7.32 ± 2.32	0.948	7.87 ± 1.14	6.53 ± 2.00	**<0.001**
av-NITBUT (s)	10.31 ± 3.03	10.83 ± 3.06	0.342	11.11 ± 1.31	9.87 ± 2.31	**<0.001**
TMH (mm)	0.37 ± 0.07	0.39 ± 0.06	0.320	0.42 ± 0.05	0.37 ± 0.07	**<0.001**
Loss on upper eyelid meibography (%)	18.00 ± 8.44	17.62 ± 8.43	0.804	15.28 ± 3.03	21.24 ± 9.55	**<0.001**
Loss on lower eyelid meibography (%)	19.32 ± 8.25	19.70 ± 8.80	0.806	17.09 ± 3.80	21.96 ± 8.90	**<0.001**

Comparison among groups was calculated using unpaired *t*-test and chi-square exact test. The comparisons with statistical differences were marked in bold.

### The association between sleep quality and dry eye disease-related parameters in the obstructive sleep apnea cases

**FIGURE 2:**
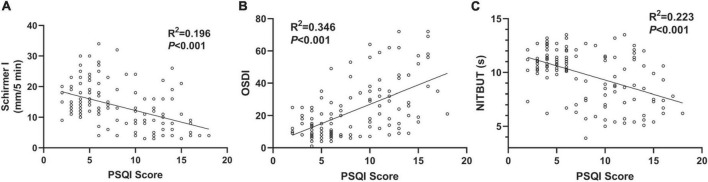
Associations between PSQI score and DED-related parameters. **(A)** A negative correlation between PSQI score and Schirmer I test value in the OSA cases (*R*^2^ = 0.196, *P* < 0.001). **(B)** A positive correlation between PSQI score and OSDI score (*R*^2^ = 0.346, *P* < 0.001). **(C)** A negative correlation between PSQI score and NIBUT (*R*^2^ = 0.223, *P* < 0.001). All the trend lines were simulated with a linear regression model.

As significant differences were detected in DED-related parameters, potential correlations were also found between PSQI scores and DED-related parameters. Among all the DED-related parameters, three parameters, including the Schirmer I test, OSDI score, and NIBUT, were selected for linear correlation analyses. As shown in Figure 2A, a negative correlation was found between the PSQI score and Schirmer I test value (*R*^2^ = 0.196, *P* < 0.001). Besides, a positive correlation was demonstrated between PSQI score and OSDI value considering the clinical symptoms (*R*^2^ = 0.346, *P* < 0.001, Figure 2B). NITBUT was regarded as an index for tear film quality and was also reported to be associated with an increased PSQI score (*R*^2^ = 0.223, *P* < 0.001, Figure 2C).

**FIGURE 3:**
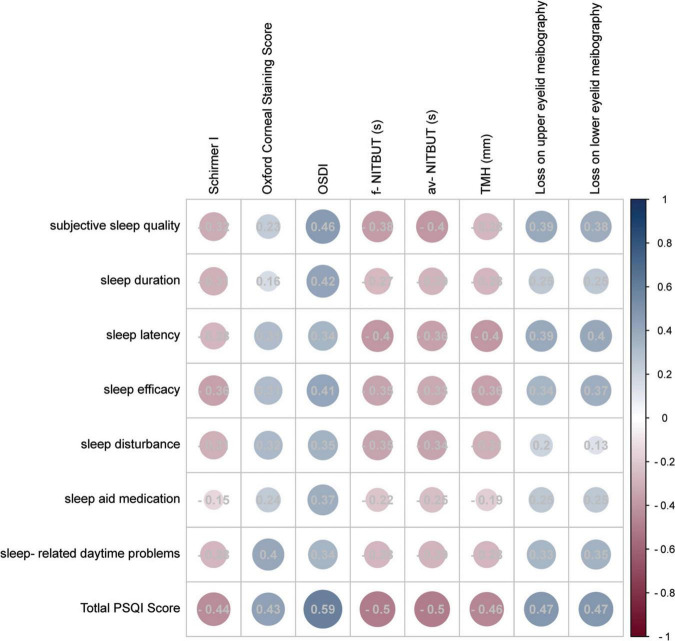
Correlation matrix of the subscales of PSQI and DED-related parameters. The circle size reflected the Pearson *R*-value, and detailed Pearson’s *R*-values were recorded in the circles. The color reflected the correlation coefficient and most red reflected −1 while most blue reflected 1.

To gain a better understanding of the association between sleep quality and DED-related parameters, the correlations were analyzed between detailed PSQI contents and all DED-related parameters, which are presented in Figure 3. An insignificant difference was only detected in the correlation between sleep aid medication and Schirmer I test values (*P* = 0.086). In the analysis of all correlation matrices between sleep quality scales and DED-related parameters, OSDI demonstrated the most significant correlation index (*R* = 0.34 to 0.59, *P* ≤ 0.001).

## Discussion

Current observational studies demonstrated that OSA, sleep disorders, and DED are all prevalent disorders. However, the detailed relationship between these disorders and their potential biological mechanisms remains unclear. In this case–control study, DED and poor sleep quality were found to be more prevalent in OSA cases. Further classification of the OSA cases into mild, moderate, and severe groups showed that the DED incidence was found to be higher in the severe OSA group. Moreover, poor sleep quality was related to a higher risk of DED among all the participants, while worse sleep quality was related to severe DED. The analysis of the relationship between PSQI contents and DED-related parameters showed that PSQI scale contents were positively correlated with the OSDI score. Our study highlighted the potential role of sleep quality in the relationship between OSA status and DED risk.

Obstructive sleep apnea can reduce the quality of life, leading to multi-system disorders, including cognitive function impairment, systemic hypertension, cardiovascular diseases, and metabolic syndromes (19). Even several therapies, including surgery, mandibular advancing device (MAD) and continuous positive airway pressure (CPAP), have been applied in OSA management (20, 21), and several accompanying diseases should be noticed. Recent studies illustrated that DED may be a comorbidity of OSA. This finding may provide an updated understanding of both pathological mechanisms and disease management. In a recent hospital-based cross-sectional study, OSA was found to be a risk factor for DED (OR = 4.36, 95% CI: 1.26 to 17.08) (22). As indicated in our results, the OSA cases showed a higher risk of DED compared to the age–gender controls, which was consistent with the previous studies. Since the association between DED and OSA incidence has been indefinite, this study can provide new evidence on the topic. Moreover, our results demonstrated the incidence of DED and dysfunctions in DED-related parameters in severe OSA cases, suggesting the induction of DED risk by severe OSA status. Our results also illustrated that severe OSA might induce further pathological changes, indicating its involvement in the potential direct or indirect pathological mechanisms underlying the incidence of DED.

Although the association between OSA and DED incidence was revealed in previous studies, there is a need for further detailed knowledge of the contributions of OSA toward the risk of DED. Among all DED subtype classifications, aqueous-deficient and evaporative DED subtypes are the most common ones (23). Generally, aqueous-deficient DED is related to reduced tear production, while evaporative DED is related to an abnormal lipid layer, which includes MGD. In the current study, the aqueous-deficient DED-related parameter, namely TMH, showed no significant difference between control and OSA groups at different stages. However, the severe OSA cases showed lower Schirmer I test values, suggesting an increased risk of aqueous-deficient DED. Moreover, floppy eyelid syndrome was also a most frequently reported comorbidity of OSA, which affects tear film dynamics and contributes to a high rate of water evaporation (24, 25). These results demonstrated that OSA might contribute to the incidence of aqueous-deficient DED; however, well-designed studies may be required in future. Considering the evaporative DED-related parameters, NITBUT and meibography were found to be significantly impaired in the OSA cases, suggesting the association of OSA with MGD-related evaporative DED. Several previous studies have reported a relationship between OSA and morphological alterations in the meibomian gland, while two independent case–control studies have highlighted the potential contributions of OSA in MGD-related DED (8, 26). Although the current study, along with other studies, has reported the relationship between OSA status and morphological alterations in the meibomian gland, the detailed biological mechanisms remain unclear. The physiological characteristics of OSA include partial or complete chronic upper airway obstruction, leading to intermittent hypoxia, sleep fragmentation during sleep, induced sympathetic nerve activation, oxidative stress, hypercoagulable state, inflammation, endothelial function damage, and metabolic abnormality (27). Intermittent hypoxia can lead to the production of reactive oxygen species, oxidative stress, endothelial function damage, and an increase in the activity of pro-inflammatory cytokines, adhesion molecules, and procoagulant factors. This leads to systemic inflammation through a series of transduction pathways (28). Inflammation plays an important role in the occurrence of both OSA and DED, with important inflammatory mediators in DED being interleukin 6 (IL-6) and C-reactive protein (CRP) (29). OSA is independently associated with elevated levels of CRP, IL-6, interleukin 8 (IL-8), interleukin 18 (IL-18), and tumor necrosis factor (TNF) (29, 30). In addition, the oxidative stress caused by OSA can upregulate inflammatory factors, cytokines, and adhesion molecules, in turn leading to circulatory and local inflammation. This can lead to dysfunctions of both the lacrimal and meibomian glands, thus, causing aqueous-deficient and evaporative DED, respectively. However, further clinical investigations and pathological experiments are required for a better understanding of the exact role of OSA in DED risk.

Obstructive sleep apnea is one kind of sleep disorder causing poor quality of sleep (31). Several recent studies reported the association between sleep quality and DED status. In a cross-sectional study, poor sleep quality was reported to be associated with the symptoms of DED (4). Several other independent studies have also reported the potential association between sleep quality and DED severity (12, 32, 33). In this case–control study, only the OSA group showed a relationship between poor sleep quality and higher DED risk and not the control group. The differences in the distribution of DED risk between the OSA and control groups indicated that poor sleep quality might contribute to the incidence of OSA-related-DED. In this observational study, we explored the correlations between all the subscales of PSQI for sleep quality analyses and DED-related parameters and interestingly found that OSDI scores were the most significant among all DED-related parameters. However, as the OSDI scale is a commonly used international questionnaire to quantify the subjective symptoms of DED, potential overlaps can be observed between DED-related clinical symptoms and sleep quality-related discomforts. Also, since ocular discomfort scales are considered an important tool for primary care practice and earlier recognition, our study suggests further attention to the usage of the OSDI scale in OSA-related DED management.

The data in this study provided evidence of correlations between OSA status and DED risk and also proposed a hypothesis that poor sleep quality can contribute to the DED incidence in OSA cases. However, this study had several limitations. First, the amount of included participants was not large enough, and a deeper understanding may be needed through well-advanced and-designed studies with a larger population. Second, DED, OSA, and poor sleep quality are complex disorders, and understanding their interactions thoroughly is difficult. Although the preliminary conclusion in the current study revealed that poor sleep quality contributed to OSA-related DED incidence, further observational studies may be required. Third, we did not consider surgery, the usage of CPAP, or other nasal mask therapy devices in the treatment of OSA nor considered their effects on DED management. These devices are important since they may aggravate DED in severe OSAS cases. A contradictory conclusion on CPAP therapy in DED incidence was reported in previous studies. A previous study demonstrated a higher DED prevalence in patients with CPAP or other nasal mask therapy (34); however, another study reported that long-term (at least 1 year) use of CPAP improved the tear quality and overcame the ocular discomforts encountered in the early stage of CPAP (35). The exact effect of OSA treatment on the risk of DED needs further investigation in well-advanced studies. Finally, the confounding bias existed in the correlation between the OSA indexes and DED-related parameters. An ongoing study with more participants and relatively simple DED-related assay was conducted in our team and thus better statistical analyses using multiple regression analyses.

## Conclusion

In conclusion, the OSA cases showed a higher prevalence of DED, while the severity of OSA was significantly correlated with the impairments in DED-related parameters. Moreover, sleep quality was found to be correlated with the onset of both OSA and DED, where poor sleep quality revealed a relationship between OSA and DED risk. Our study results suggest that clinicians engaged in DED and OSA management should pay attention to the interaction between DED and OSA. However, further investigations may be required to study the effects of sleep quality on the risks of DED and OSA, providing an understanding of both biological mechanism detection and clinical disease management. Moreover, further well-designed clinical studies may be required to analyze the relationships between OSA status, DED incidence, and sleep quality to provide better insights into improved therapeutic strategies.

## Data availability statement

The original contributions presented in this study are included in the article/Supplementary material, further inquiries can be directed to the corresponding author.

## Ethics statement

The studies involving human participants were reviewed and approved by Changshu No. 2 People’s Hospital Medical Ethics Committee. The patients/participants provided their written informed consent to participate in this study.

## Author contributions

X-YL: supervision, project administration, term, conceptualization, and writing—review and editing. QP and ZW: investigation, formal analysis, visualization, and writing—original draft preparation. A-LL, X-XG, and J-JH: data curation and validation. All authors contributed to manuscript revision, read, and approved the submitted version.

## Supplementary material

Supplementary Figure 1 Image of TMH. TMH is the plane height formed by tears, margin of inferior eyelid and inferior bulbar conjunctiva, which can reflect the amount of lacrimal gland secretion. The normal value of TMH is 0.4∼1 mm, and TMH less than 0.35 mm indicates the possibility of DED.

Supplementary Figure 2 Image of meibomian gland. **(A)** Image of upper meibomian gland, and the loss of upper meibomian gland was 58.4%. **(B)** Image of lower meibomian gland, and the loss of lower meibomian gland was 5%.
