# Supplementary figures and images for: Association between poor sleep quality and an increased risk of dry eye disease in patients with obstructive sleep apnea syndrome
Source: Front Med (Lausanne). 2022 Oct 31;9:870391. doi: 10.3389/fmed.2022.870391 (PMC9659957; doi:10.3389/fmed.2022.870391)

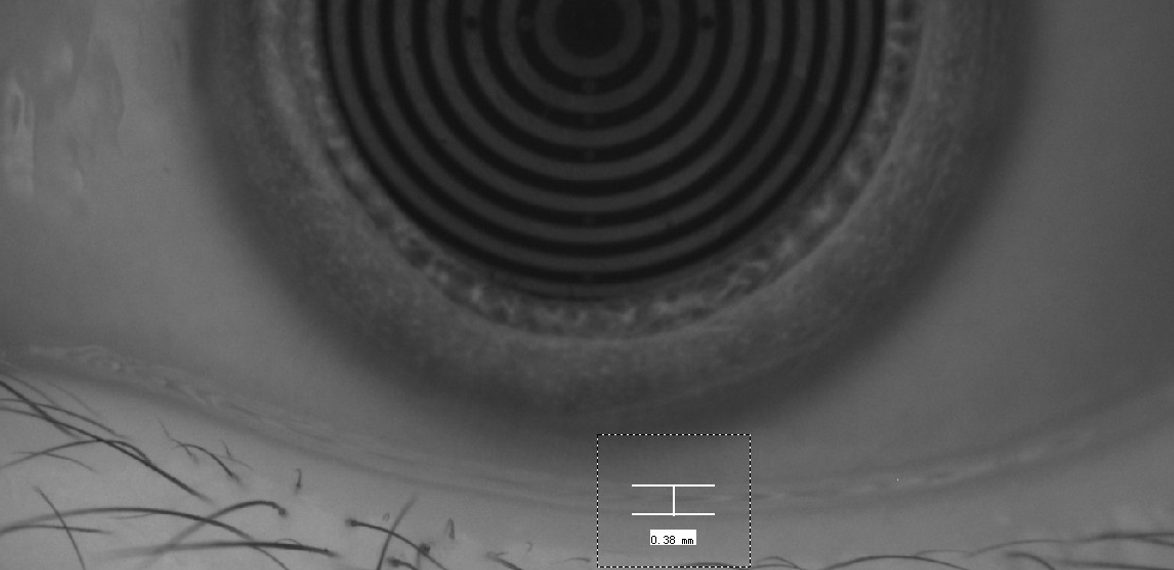

Supplement: Supplementary Figure 1 — Image of TMH. TMH is the plane height formed by tears, margin of inferior eyelid and inferior bulbar conjunctiva, which can reflect the amount of lacrimal gland secretion. The normal value of TMH is 0.4∼1 mm, and TMH less than 0.35 mm indicates the possibility of DED. [file Image_1.tif]

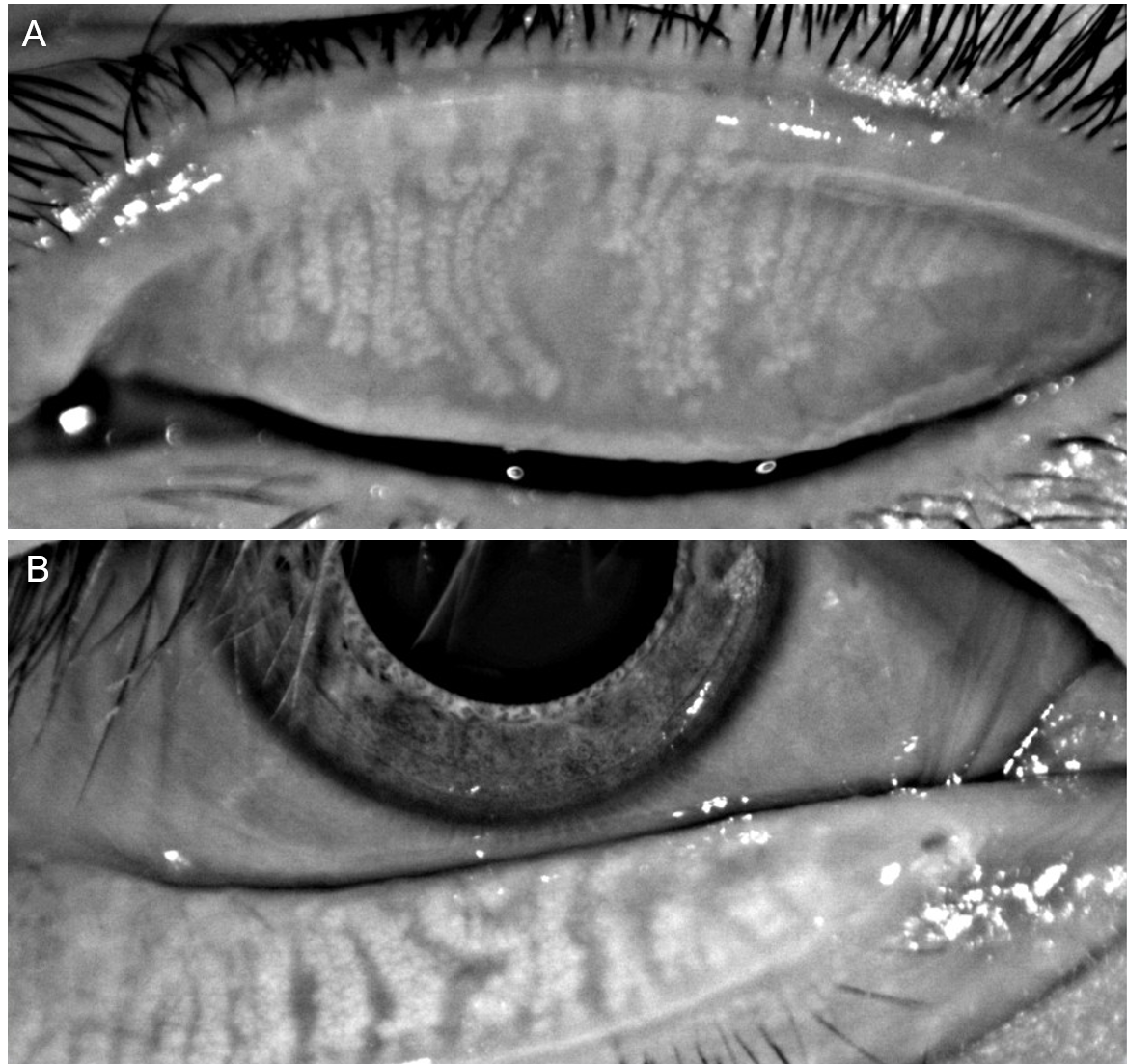

Supplement: Supplementary Figure 2 — Image of meibomian gland. (A) Image of upper meibomian gland, and the loss of upper meibomian gland was 58.4%. (B) Image of lower meibomian gland, and the loss of lower meibomian gland was 5%. [file Image_2.tif]
